# Nanocurcumin and curcumin prevent N, N'-methylenebisacrylamide-induced liver damage and promotion of hepatic cancer cell growth

**DOI:** 10.1038/s41598-022-12406-y

**Published:** 2022-05-18

**Authors:** Mona M. Atia, Hanem S. Abdel-Tawab, Amna M. Mostafa, Seham A. Mobarak

**Affiliations:** 1 Laboratory of Molecular Cell Biology and Laboratory of Histology, Zoology Department, Faculty of Science, Assiut University, Assiut, 71516 Egypt; 2 Department of Zoology, Faculty of Science, South Valley University, Qena, Egypt

**Keywords:** Cancer, Cell biology

## Abstract

Acrylamide (AC) is an environmental contaminant with cancer-promoting and cytotoxic properties, while curcumin (Cur.) is a phytochemical with documented anticancer and cytoprotective efficacy. Nanoparticle formulations can increase the efficacy of phytochemicals, so we examined the anticancer and hepatoprotective efficacies of nanocurcumin (N.Cur). Curcumin and nanocurcumin reduced HepG2 and Huh-7 cancer cell viability and increased apoptosis in the presence and absence of AC, while AC alone promoted proliferation. Furthermore, the anticancer efficacy of nanocurcumin was greater than that of curcumin. In mice, AC greatly increased hepatic expression of CYP2E1, P53, cleaved caspase-3, and COL1A1 as well as serum alanine aminotransferase and aspartate aminotransferase activities. These effects were reversed by nanocurcumin and curcumin. Nanocurcumin also reduced the histopathology and fibrosis caused by AC, and reversed AC-induced glycogen depletion. Nanoparticle formulation can increase the anticancer and hepatoprotective efficiencies of curcumin.

## Introduction

Acrylamide (AC) is a synthetic compound widely used in industry^1^ and generated by certain manufacturing processes that are now regarded as an environmental contaminant of concern due to documented systemic toxicity^2^. Moreover, acrylamide is now classified by the International Agency for Research on Cancer as a probable human carcinogen^3^. As a small unsaturated amide, AC is absorbed readily by humans and animals after ingestion and distributed into several vital organs such as the thymus, heart, brain, liver, and kidney, where it may induce toxicity or carcinogenicity^4^.

Once ingested, AC is conjugated with glutathione via an oxidation reaction driven by cytochrome CYP2E1 or cellular glutathione-S-transferases, which involves oxidation of AC to its epoxide derivative, glycidamide (GA). Both AC and GA react with nucleophilic sites in macromolecules (including HB and DNA) in Michael-type additions^5,6^. Acrylamide present in foods has the potential to provoke pro-inflammatory states in the body and enhance atherosclerosis risk^7^. Also, acrylamide causes oxidative damage to DNA and the collapse of mitochondrial membrane potential (MMP)^8^. Wide attention and concern about human toxicity from AC exposure arises from the observations that AC is neurotoxic in experimental animals and humans, mutagenicity in somatic, and germ cells and its carcinogenicity to several organs^9^.

The bioavailability and efficacy of these chemopreventive agents can be enhanced by incorporation into nanotechnology-based drug delivery systems. Indeed, curcumin nanocrystal conjugates have demonstrated improved stability and efficacy as natural therapeutic agents^10^. In the mouse brain, for example, nano- curcumin demonstrated superior bioavailability and antioxidant efficacy compared to curcumin^11^. Therefore, curcumin nanoparticles with even greater efficacy could have broad applications in clinical medicine. N.Cur enhanced and lowered caspase-3 and Bcl-2 protein expression in HepG2 cells, making them more susceptible to apoptosis^12^. Despite cur. and N.Cur.having the identical chemical structure, N.Cur. has a more effective antibacterial effect than Cur^13^.

Treatment with N-Cur. also lowered the levels of oxidative stress indicators and raised antioxidant content in the tissues^14^. Fascinatingly, N.Cur supplementation prevented liver enzymes (AST, ALT) from rising^15^. N.Cur hinders profibrogenic transcripts linked with myofibroblasts, and mouse hepatic fibrosis^16^. Curcumin can induce apoptosis via tumor suppressor protein p53-related signaling pathways of many hepatic cancer cell lines, suggesting great potential in hepatocellular carcinoma prevention and therapy^17^. Further, curcumin has demonstrated antioxidant, antibacterial, anti-inflammatory, anti-aging, and anticancer properties^18^ and HepG2 cell growth and metastasis^19^.

The main goal of the current study was to create nano-sized curcumin (nano-curcumin) and to assess the potential protective efficacy of this formulation against acrylamide-induced oxidative stress and its toxicity against liver and cancer cells.

## Material and methods

### Materials

Acrylamide and Curcumin (Sigma-Aldrich), mouse monoclonal IgG anti-p53 (DO-1) antibody, goat polyclonal IgG anti-cleaved Caspase 3 (P11) antibody, mouse monoclonal IgG anti β actin (2A3) antibody, goat anti-mouse IgG-HRP (sc-2031), mouse anti-rabbit IgG-HRP (sc-2357) and mouse anti-goat IgG-HRP: sc-2354 were from Santa Cruz Biotechnology (Dallas, TX, USA). Rabbit polyclonal IgG anti-Cytochrome P450 2E1 (ab28146) antibody was from Abcam, Cambridge, MA, USA. These antibodies were documented to be validated for specificity by the suppliers. SYBR Green PCR Master Mix from Bio-Rad (Austria). Ethidium bromide, acridine orange, 3-(4,5-dimethylthiazol-2-yl)-2,5-diphenyltetrazolium bromide (MTT), RPMI-1640, and fetal bovine albumin (FBS) were purchased from Sigma-Aldrich (St. Louis, MO, USA). Ethylenediaminetetraacetic acid (EDTA), phosphate buffered saline (PBS), were purchased from Sigma-Aldrich (St. Louis, MO, USA). HEPES were purchased from from Thermo Fisher Scientific (Waltham, MA, USA).

### Ethics statements

This study was conducted in strict accordance with the guidelines of the National Health and Medical Research Council for the Care and Use of Animals. The Ethical Research Committee of the Faculty of Veterinary Medicine at South Valley University of Qena reviewed (No. 12/23.05.2021).

### Preparation of bis-acrylamide solution

Acrylamide was weighed and dissolved immediately in sterile distilled water immediately prior to culture application or injection^20^.

### Preparations of nanocurcumin

N.Cur. was synthesized from a 5 mg/ml solution of raw Cur. in dichloromethane (100 mg curcumin powder was dissolved in 20 ml dichloromethane) as reported^21^.

### Morphological analysis of N.Cur. by transmission electron microscope (TEM)

A drop of nanocurcumin (20 nm/100 g/l) was dropped onto a carbon-coated copper grid and allowed to dry at room temperature. The transmission electron microscope was used to take TEM micrographs of this sample (The size and shape). Analyses were carried out utilising a high-resolution transmission electron microscope (HR-TEM, JOEL JEM-2100) with a Gatan digital camera and operating at a 200 kV accelerating voltage. A drop from a very dilute sample solution was deposited on an amorphous carbon coated-copper grid and left to evaporate at room temperature. The TEM images were recorded and the lattice spacing values calculated from the electron diffraction pattern. (Assiut University, Faculty of Science, Chemistry Department).

### Analysis of N.Cur. by dynamic light scattering (DLS) and absorbance properties

Chemical absorbance properties were examined by UV–visible spectroscopy^22^. A Malvern Zetasizer ZS (Malvern Instruments, UK, Nawah-Scientific Egypt) was used to measure the size distribution and surface zeta potential of N.Cur. by dynamic light scattering (DLS)^23^. All characterization measurements were carried out three times.

### Ethical approval

I confirm, the study of our manuscript is reported in accordance with ARRIVE guidelines.

## Cancer cell maintenance and treatments

### Cell culture

HepG2 and Huh7 cancer cell lines (Nawah-Scientific, Egypt) were cultured in RPMI-1640 growth medium supplemented with 1% antibiotic mix (10.000 u penicillin/ml, and 10.000 u streptomycin/ml) and 10% FBS at 37 °C under an atmosphere of 100% humidity and 5% CO_2_.

### Treatment

HepG2 and Huh7 cell lines were seeded in 96-well plates at 2 × 10^3^ cells/well with RPMI plus 10% heat-inactivated FBS and maintained at 37 °C under 100% humidity and 5% CO_2_ during treatment. Cur. and N.Cur. stock solutions were prepared at 20 mM in DMSO and kept at − 20 °C. Cells were treated with 20 µM Cur., 20 µM N.Cur.,^24^ and/or 10 µM acrylamide (in DMSO) as indicated for 24 or 48 h. Control cells were cultured in the same medium containing equal-volume vehicle.

### MTT assay

After drug treatment, growth medium was exchanged for 200 µL of drug-free medium and 50 µL of MTT (2 mg/ml). The formazan produced from MTT by viable cells during 3 h of incubation was dissolved in 200 µL DMSO and absorbance measured at 570 nm and reference wavelength of 630 nm as an estimate of viable cell number. The results are expressed as a percentage of vehicles (DMSO)-treated control cell number^25^.

### Acridine orange/ethidium bromide (EB/AO) assays

Cells suspensions (2.0 × 10^6^ cells/ml) treated as indicated were assayed for apoptosis by acridine orange/ethidium bromide uptake. Briefly, treated cells were washed in 200 µL of warm 1X PBS, resuspended in 150 µL AO/EB dye for 5 min, rinsed in 400 µL of 1X PBS, pelleted by centrifugation at 277×*g* for 2 min at room temperature, resuspended in PBS, and viewed immediately under a fluorescence microscope equipped with a fluorescein filter and 20× objective. Samples were mounted with moviol/DABCO and examined under Olympus BX41 fluorescence microscope at 20X objective length with NA 0.40 (200X magnification) with 480/30 nm excitation filter and 535/40 nm emission filter. Images were captured using Toup Tek ToupView, Copyright© 2019, Version: × 86, Compatible: Windows xp/Vista/7/8/10, China.

### Animal experiments and treatments

Fifty healthy male Swiss albino mice (*Mus musculus*) weighing 25–30 g was purchased from the animal house Theodor bilharziainstitute (Giza, Egypt). Mice were divided into five groups, control, vehicle control, AC, AC + Cur, and AC + N.cur, and the indicated drugs were administered daily for four weeks. The control group received distilled water, the vehicle control group Tween-80, the AC group 3 mg/kg oral AC^21^, and the AC + Cur and AC + NCur groups 7 mg/kg oral Cur. or N.Cur.^26^ 30 min prior to 3 mg/kg oral AC.

### Western blot analysis

Changes in hepatic cytochrome P450 (CYP2E1) and P53 were measured by immunoblotting as described^27^. Liver samples were lysed by homogenizer at 4 °C in 500 µl of RIPA lysis buffer (1% Nonidet-P40, 1% Triton X-100, 0.5% Na deoxycholate, 150 mM NaCl, 1 mM PMSF, 5 mM EDTA, 10 mM EGTA, 50 mM Tris–HCl, and 1% leupeptin/pepstatin protease inhibitor cocktail). Centrifugation at 10,000×*g* for 10 min at 4 °C was used to remove insolubilized tissue debris. The protein concentration in the supernatant was measured. SDS-PAGE (10%) was utilized to resolve protein aliquots, which were transferred onto a nitrocellulose membrane. The membranes were blocked with 5% skim milk in TBS containing 0.05% Tween 20 and then incubated with primary antibodies anti P53 and P450 (1:1000) overnight at 4 °C. Subsequently, the membranes were incubated with the suitable HRP-conjugated secondary antibodies (1:10,000) in the blocking solution for 1 h at 24 °C. Chemiluminescent substrate kit was used to see the immunoreactive bands. For equal loading confirmation, an anti-actin goat polyclonal antibody was utilized. The data are expressed as mean ± SE from at least three separate experiments; the statistical software version of Fiji/Image J was used to estimate each band's optical density in relation to the corresponding actin band.

### Tissue immunohistochemistry

Liver tissues were embedded in paraffin, sectioned at 3 µm, deparaffinized, rehydrated in gradient ethanol (100–70%), heated in 10 mM sodium citrate buffer (pH 6.0) for antigen retrieval, developing sections in 3% H_2_O_2_ for 10 min, washing with wash buffer (1X PBS) for 5 min, and then blocking each section at room temperature with 100–400 µL blocking solution for 1 h. the cleaved caspase 3 primary antibody was added (1:10) after the blocking solution was removed. The sections were then treated with secondary antibody (1: 5000) for 2 h, washed, stained with 3, 3′-diaminobenzidine (DAB) for 2–3 min, and counterstained with hematoxylin The reaction was immediately quenched in distilled water. A light microscope was used to visualize the stained sections^28^.

### Quantitative real time RT-PCR

Liver tissue was homogenized and total RNA isolated using Triazole reagent (Invitrogen). RNAs were reverse transcribed using the High-Capacity cDNA Reverse Transcriptase kit (Applied Biosystems; Thermo Fisher Scientific, Inc.). Quantitative RT-PCR was then performed in 25 µL reaction mixtures containing 1 µL template cDNA, SYBR green PCR Master mix (Applied Biosystems), 10 pmol of Primers for collagen type I alpha 1 (COL1a1) (**forward,** TCTGCGACAACGGCAAGGTG and **reverse,** GACGCCGGTGGTTTCTTGGT) and for GAPDH as the internal control (**forward,** AAC TTT GGC ATT GTG GAA GG and **reverse,** GTC TTC TGG GTG GCA GTG AT)^29^.

### Liver function parameters

Alanine aminotransferase (ALT) and aspartate aminotransferase (AST) activities in blood plasma were determined using kits (Boehringer Mannheim, Mannheim, Germany) according to manufacturer procedures.

### Histopathological examination

For histological and histopathological examinations, pieces of liver tissue were fixed in 10% neutral buffered formalin (pH 7.2), dehydrated in gradient ethanol, cleared in xylene, After paraffin embedding 3–5 µm sections were mounted on glass slides. Sections were deparaffinized twice in xylol for 30 min and hydration with ethanol series then stained routinely with Hematoxylin and eosin, Picrosirius Red^30^ or Periodic acid Schiff (PAS) as indicated. Five histopathological parameters were documented, region of degeneration, cytoplasmic color fading (light or heavy eosinophilic cytoplasm), nuclear condensation, nuclear fragmentation, and inflammation^31^. Collagenous fibers were examined at 40× in randomly chosen fields from at least three animals per group, and a Fibrosis index (FI) calculated as follows: FI = Total positive area/Total section area × 100^32^.

### Statistical analysis

Data are presented as the mean ± standard error of the mean (SEM) from at least three independent experiments. Treatment group means were compared by one-way analysis of variance followed by post hoc Student Newman–Keuls *T* tests using Graph Pad Prism 3 (Graph Pad Software Inc., USA).

## Results

### Morphological and physicochemical properties of N.Cur.

**Figure 1:**
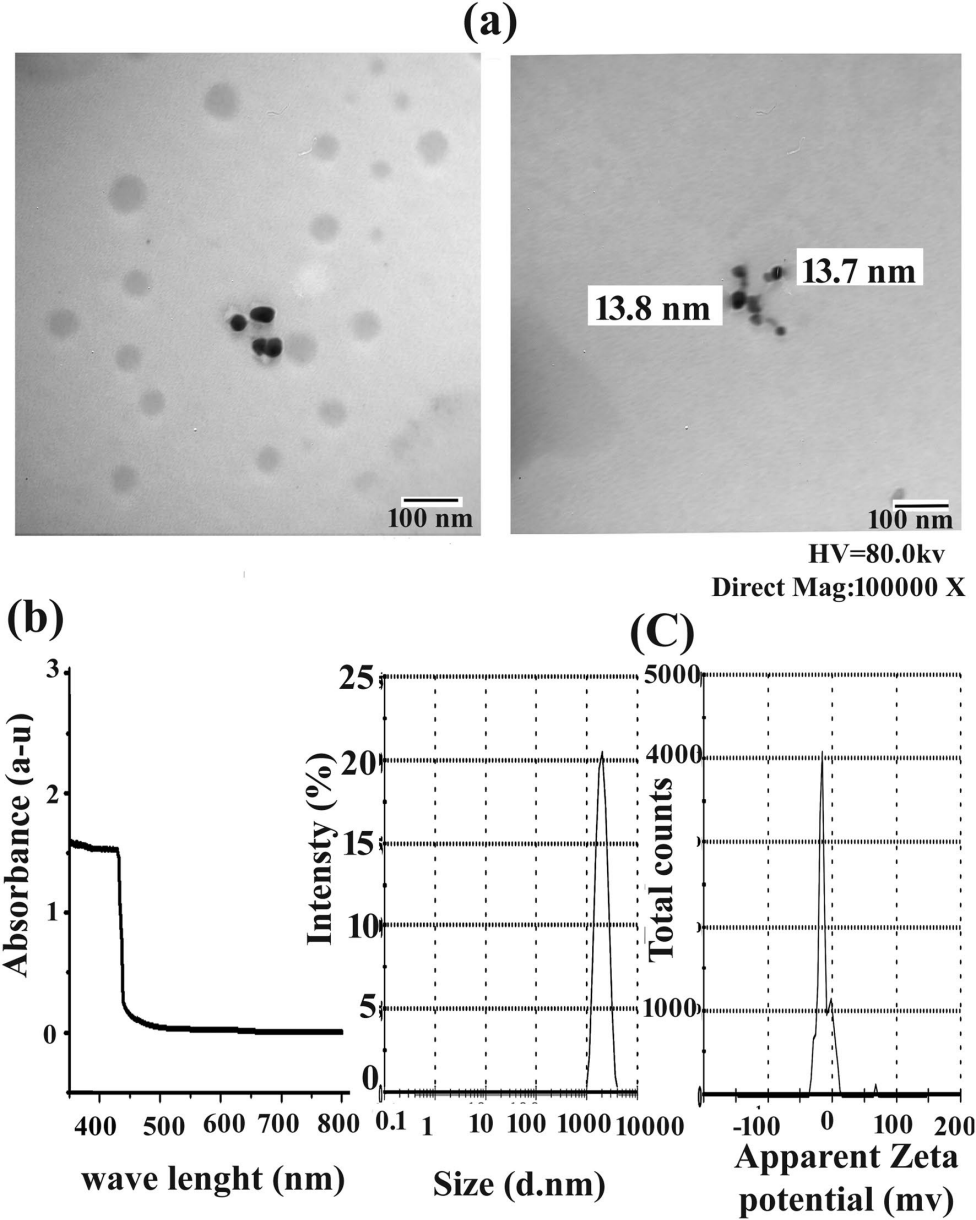
Morphology and physicochemical properties of curcumin nanoparticles. **(a)** TEM examination of nanocurcumin (N.Cur) (bar = 100 nm). **(b)** Optical absorption properties. **(c)** Measurements of zeta potential and mean particle diameter.

The size distribution of curcumin nanoparticles is shown in (Fig. 1a). Particles were of various globular forms and sizes but generally smaller than 100 nm in diameter. The absorption of N.Cur. peaked at 432 nm (Fig. 1b). The size distribution and zeta potential as determined by DLS for hydrated N.Cur. are shown in (Fig. 1c).

### Cur. and N.Cur. reduced hepatic cancer cell viability

**Figure 2:**
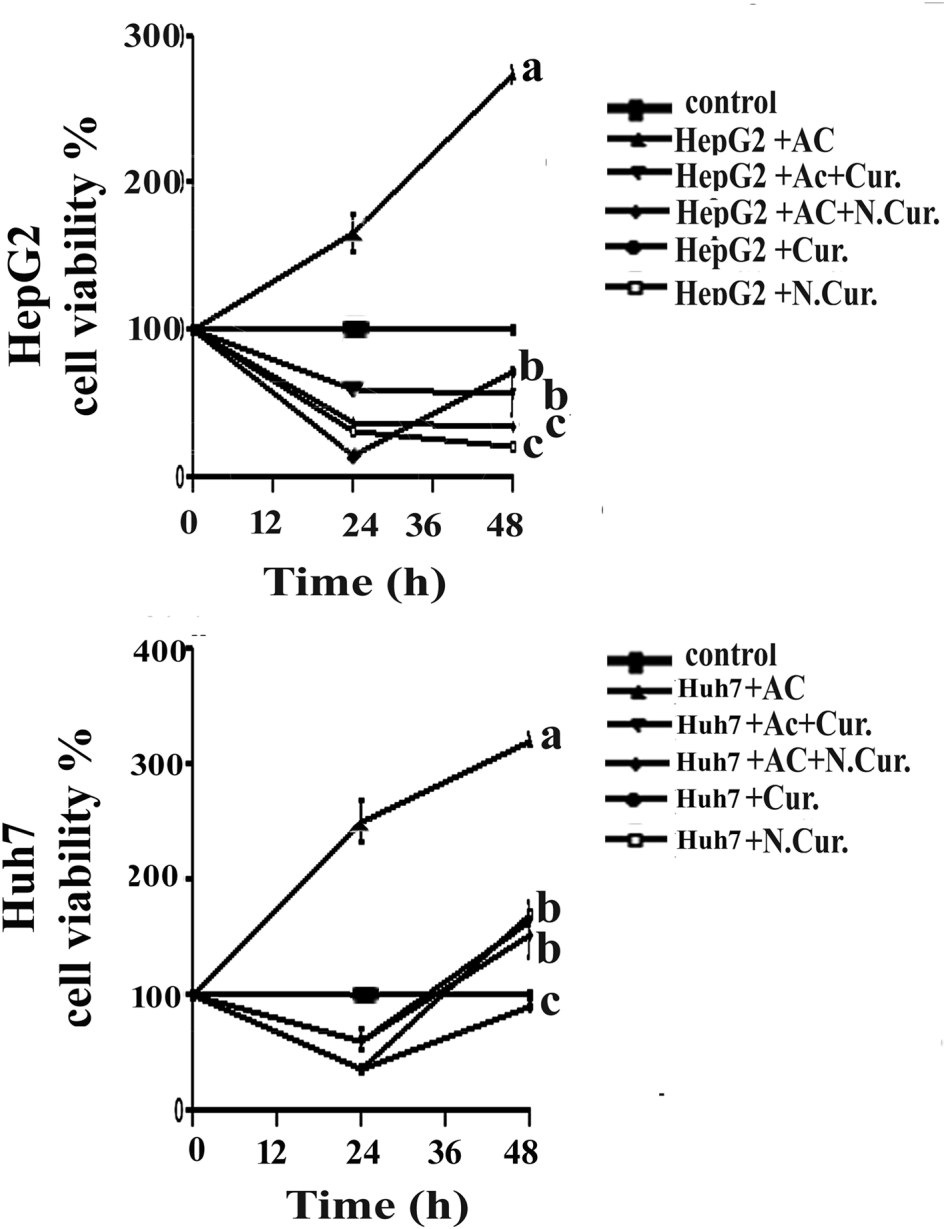
Curcumin and nanocurcumin reduced the viability of cultured HepG2 and Huh-7 hepatic cancer cells. Viability was measured by MTT assay. Data presented as mean ± SEM after 24 and 48 h of treatment. Values with unlike superscript letters are significantly different.

Both Cur. and N.Cur. demonstrated time- and dose-dependent cytotoxicity on HepG2 and Huh-7 cells as measured by MTT assay. Furthermore, in the presence of AC, there was non cytotoxicity in cell viability of HepG2 and Huh-7 cell lines after 24–48 h incubation. N.Cur. significantly reduced the cell viability and increased the cytotoxicity of both cell lines than Cur.. It was found Huh-7 cells were more resistance to N.Cur (Fig. 2).

### Cur. and N.Cur. induced hepatic cancer cell death

**Figure 3:**
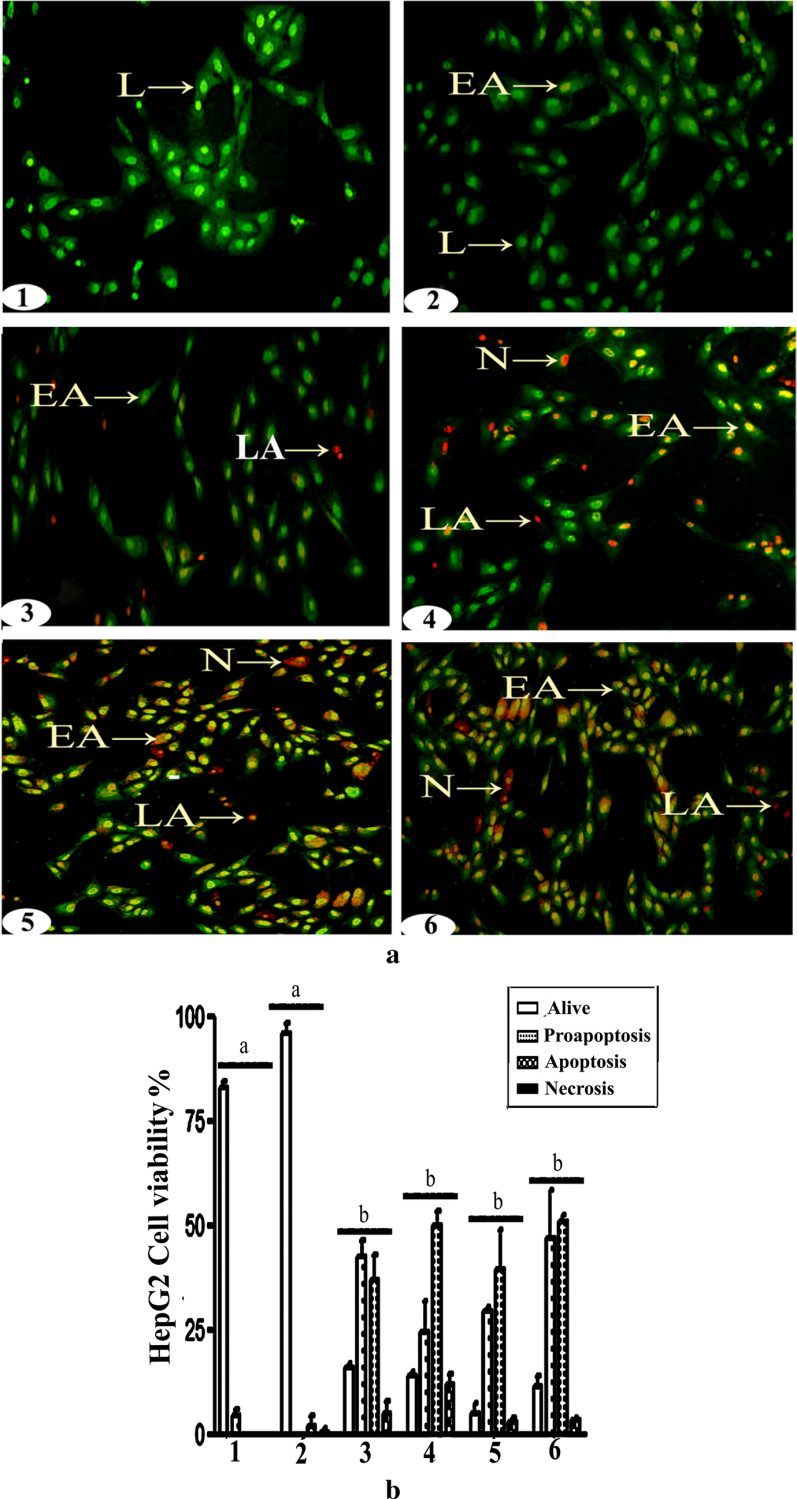
Curcumin and nanocurcumin induced apoptosis of HepG2 hepatic cancer cells. (**a**) EB/AO staining of HepG2 cells (**1** = control, **2** = AC, **3** = AC + Cur, **4** = AC + NCur, **5** = Cur, **6** = NCur) (X = 200). **(b)** Results expressed as mean ± SEM. In the following figures, L = live cells, EA = early apoptotic cells, LA = late apoptotic cells, and N = necrotic cells. Values in the same column with unlike superscript letters are significantly different at *p* < 0.001.

**Figure 4:**
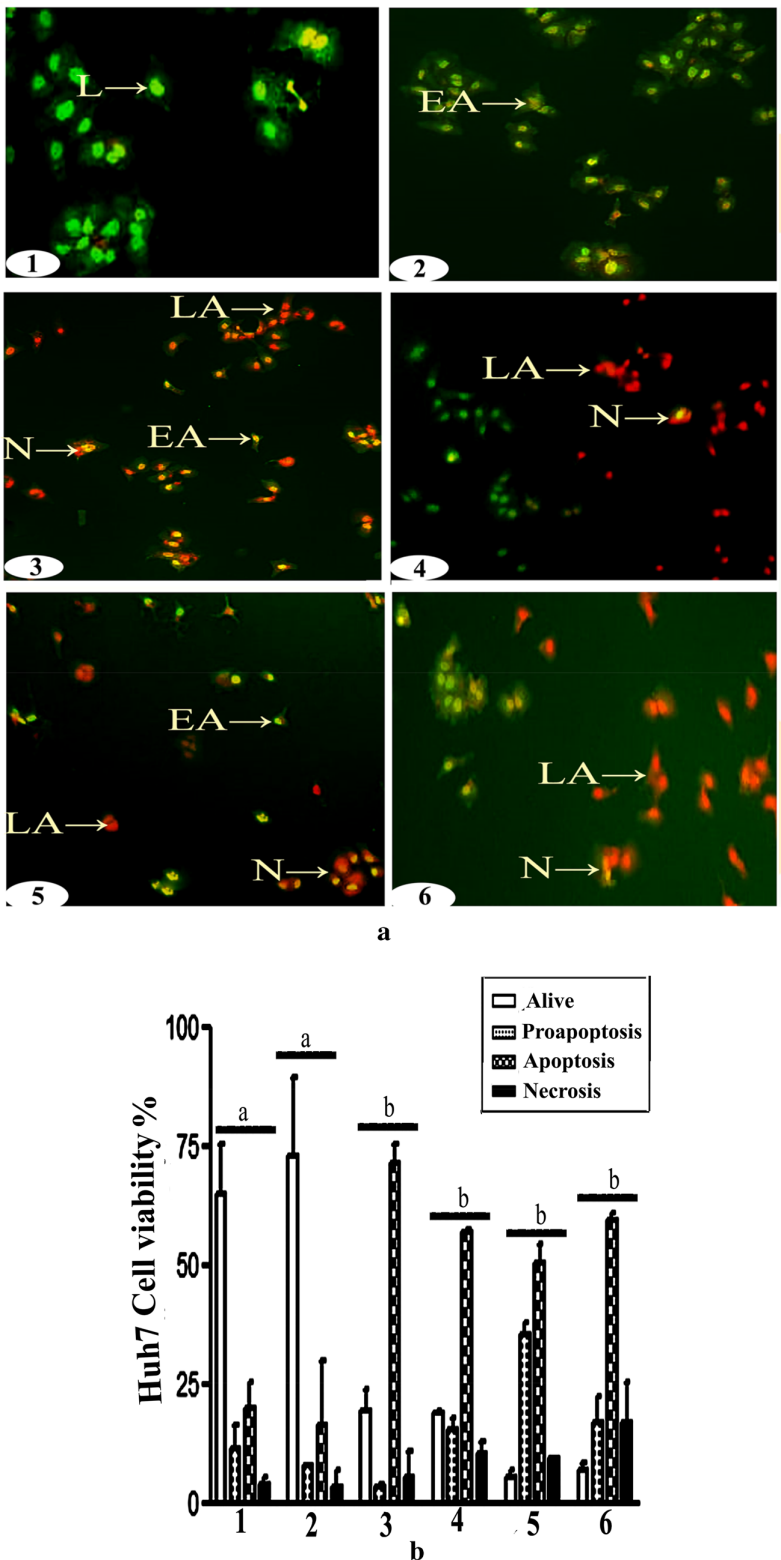
Curcumin and nanocurcumin induced apoptosis of Huh-7 hepatic cancer cells. (**a**) EB/AO staining assay of Huh-7 cells (**1** = control, **2** = AC, **3** = AC + Cur, **4** = AC + NCur, **5** = Cur, **6** = NCur). X = 200. **(b)** Results expressed as mean ± SEM. Values in the same column with unlike superscript letters are significantly different at *p* < 0.001.

In principle, these effects on viable cancer cell number could reflect inhibition of proliferation or induction of cell death. Therefore, we also conducted dual EB/AO staining for apoptosis and necrosis. Most control HepG2 and Huh-7 cells appeared to have green nuclei with intact structure. Similarly, after 24 h of incubation in AC, most HepG2 (Fig. 3a,b) and Huh-7 cells (Fig. 4a,b) were viable and no apoptotic staining was detected. However, incubation with Cur. or N.Cur. caused morphological and staining changes indicative of early apoptosis, late apoptosis, or necrosis (e.g., chromatin condensation, orange nuclei, and nuclear fragmentation). Further, N.Cur. prevented the effects of AC on proliferation and increased the proportions of cells in late apoptosis and necrosis.

### Cur. and N.Cur. reversed AC-induced upregulation of the CYP450 and tumor suppressor P53

**Figure 5:**
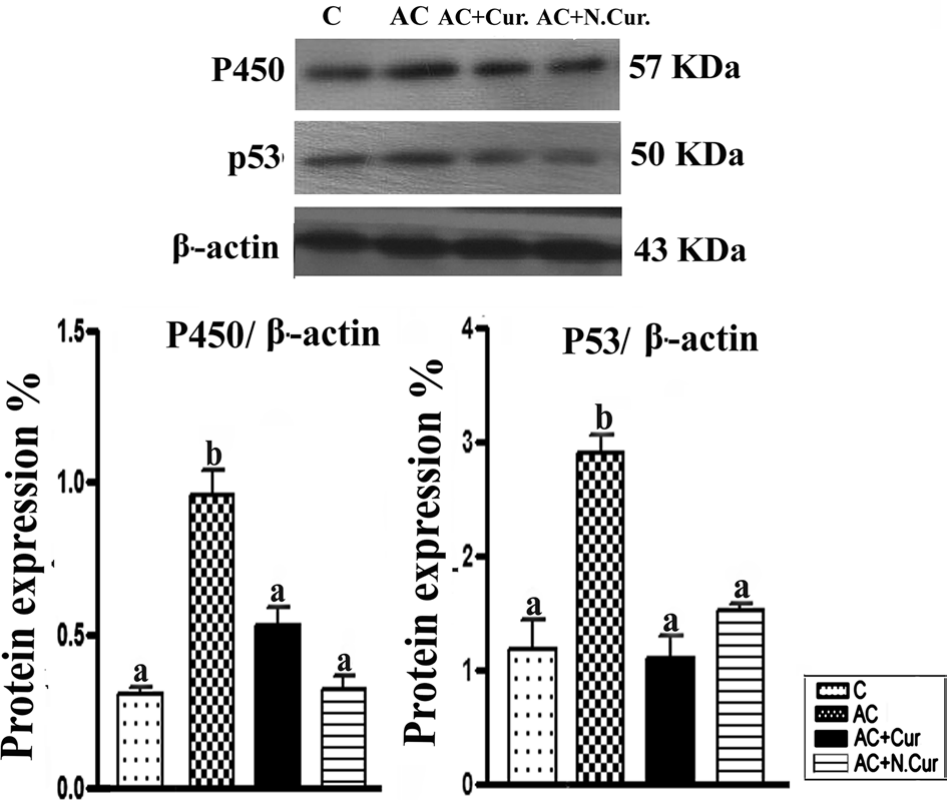
Western blot detection showing changes in protein levels of cytochrome P450 (CYP2E1) and P53. Results expressed as mean ± SEM. Values in the same column with unlike superscript letters are significantly different at *p* < 0.001.

Immunoblotting revealed that AC increased the protein expression levels of CYP450 and P53 in mouse liver compared to controls (by 216.06% and 148.37%, respectively), while co-treatment with Cur. or N.Cur. completely reversed these changes (Cur. by 44.95% and 62.36%, respectively, and. N.Cur. by 66.21% and 47.85%, respectively, versus the AC group) (Fig. 5). Thus, N.Cur. was more effective than Cur. at reducing CYP450 induction while Cur. was more effect at reducing P53 expression.

### Cur. and N.Cur. regulate apoptosis via controlling C.caspase-3

**Figure 6:**
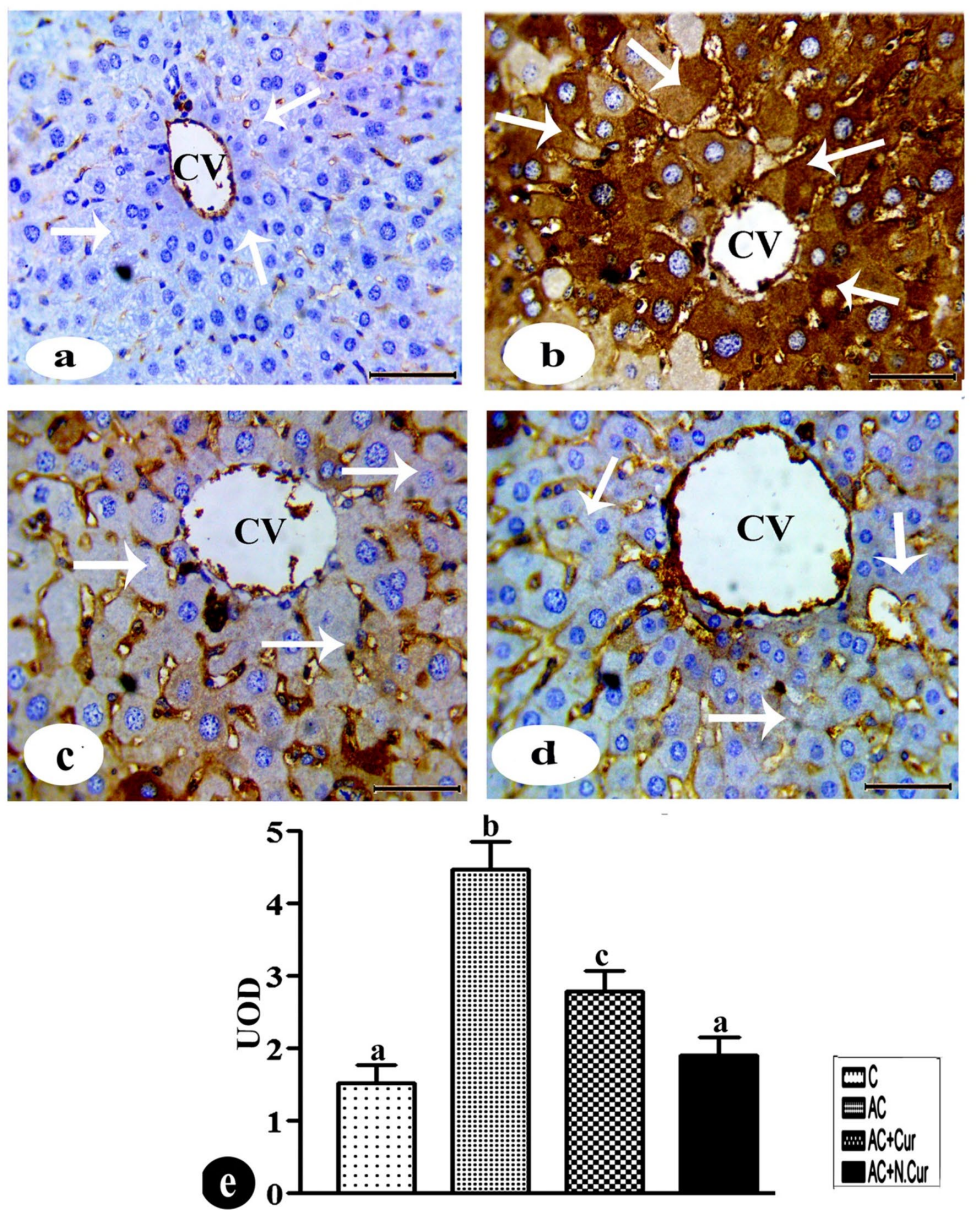
**(a)–(d)** Immunohistochemical staining for cleaved caspase-3 protein expression levels in liver tissue from **(a)** control group (negative reaction, arrow), **(b)** AC group (positive reactions, arrows), **(c)** AC + Cur group, and **(d)** AC + NCur group (negative reaction, arrows). bar = 50 µm. **(e)** Statistical, values in the column with unlike superscript letter were significantly different (*p* < 0.001).

Immunohistochemistry for the apoptosis effector cleaved revealed that AC substantially enhanced apoptotic activity in liver (by 195.2%) compared to control mice (Fig. 6a,b and e). Conversely, cleaved caspase-3 immunoexpression was downregulated by both Cur. (37.8% reduction compared to the AC group) and N.Cur. (57.5% reduction compared to the AC group) (Fig. 6c–e). Thus, nanocurcumin was more effective than curcumin at inhibiting AC-induced apoptosis in mouse liver.

### Cur. and N.Cur. reversed AC-induced upregulation of collagen expression

**Figure 7:**
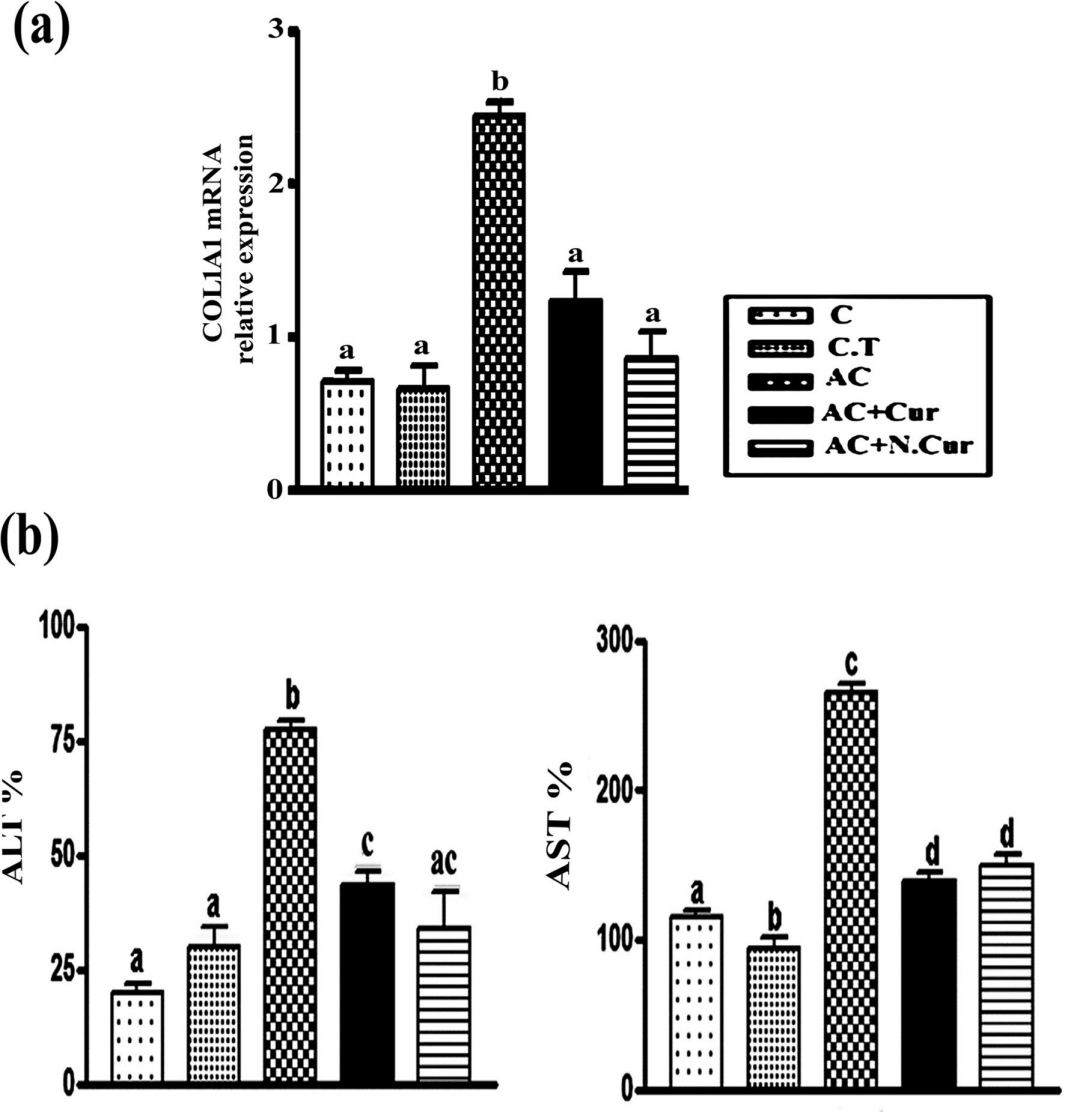
**(a)** COL1A1 expression levels (mean ± SEM). **(b)** AST and ALT levels in male mice. Values in the same column with unlike superscript letters are significantly different at *p* < 0.001.

Acrylamide administration significantly enhanced COL1A1 mRNA expression (by 248.42%) compared to control mice as measured by RT-PCR, while pretreatment with Cur. reduced expression by 49.67% and N.Cur. by 65.29% compared to AC-treated mice. Thus, both compounds may suppress AC-induced liver fibrosis, with N.Cur. showing greater efficacy (Fig. 7a).

### Cur. and N.Cur. reduced the systemic release of liver enzymes

Treatment with AC increased serum levels of AST and ALT by 130.17% and 288.57%, respectively, consistent with liver tissue damage compared to control. Consistent with hepatoprotection, however, curcumin pretreatment reduced AST by 47.68% and ALT by 43.71% compared to the AC group, while nanocurcumin reduced AST by 46.78% and AST by 56.02% compared to the AC group (Fig. 7b).

### Cur. and N.Cur. protected mouse liver from AC-induced degeneration and inflammation

**Figure 8:**
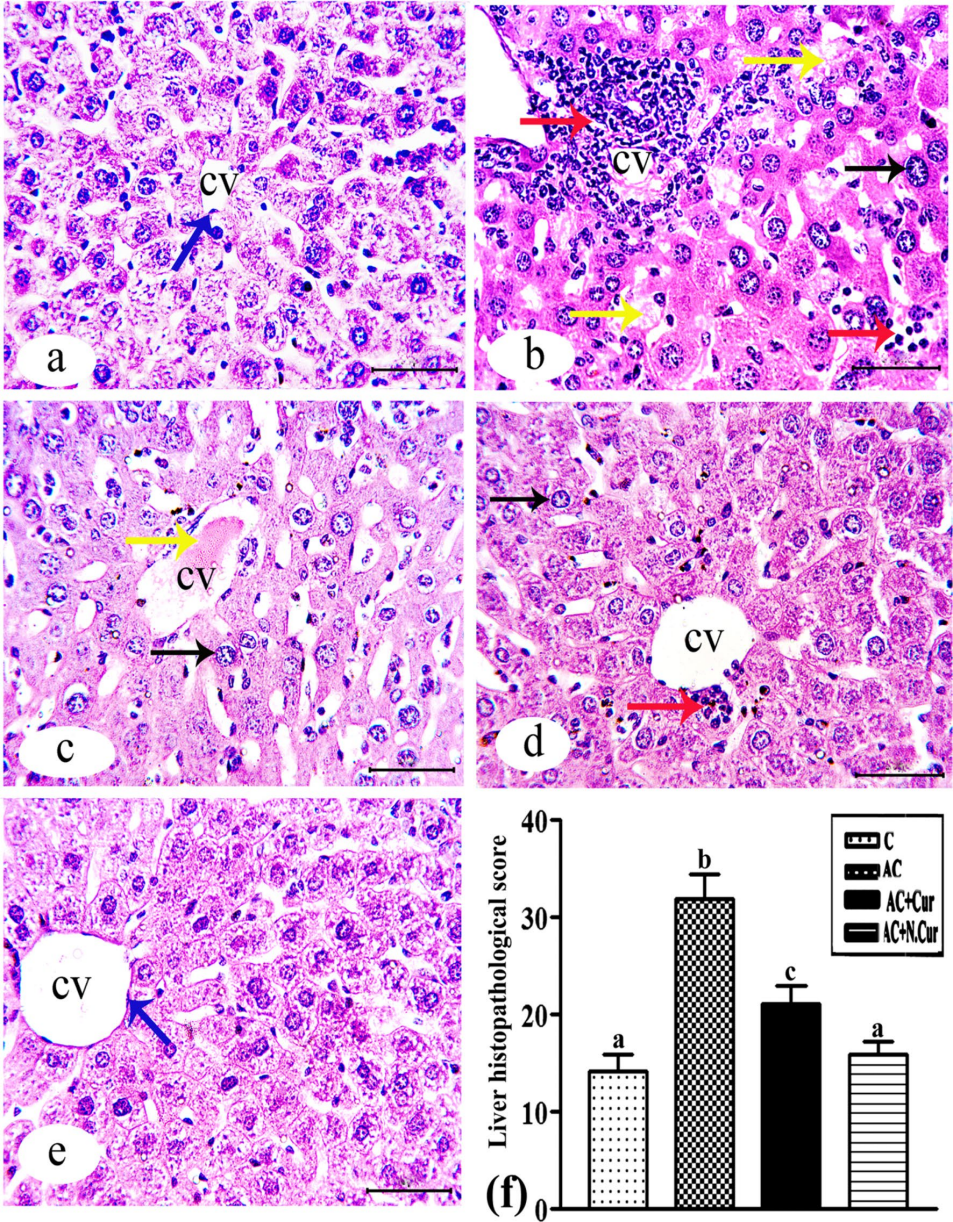
**(a)–(e)** Photomicrograph of H&E-stained liver sections from the indicated groups: **(a**) control male mice, showing the central vein (cv) and obvious appearance of Kupffer cells (blue arrow); **(b and c)** AC group mice, revealing loss of hepatic architecture, hemorrhage (yellow arrow), inflammation (red arrow), fragmented nuclei (black arrow), multiple ballooned hepatocytes, and vacuolated cytoplasm; **(d)**: AC + Cur group , showing hepatic tissues with more modest cell infiltration (red arrow); **(e)**: AC + NCur group, demonstrating healthy architecture appearance similar to control (H&E Bar = 50 µm). **(f)** Liver histopathology scores of groups (mean ± SE) and values with different letters are significantly different (*p* < 0.001).

Liver sections from control mice exhibited typical hepatic tissue architecture as revealed by HE staining (Fig. 8a), while AC treatment induced focal necrosis, pyknotic nuclei, inflammatory leucocytic infiltration, and the formation of lipid droplets (Fig. 8b). In some specimens, hepatocytes exhibited giant nuclei or vacuolization indicative of hydropic degeneration, while blood sinusoids were dilated and the central vein showed signs of congestion (Fig. 8c). Pretreatment with Cur. reduced the number of lipid droplets as well as the number and size of focal necrotic areas (Fig. 8d), while N.Cur. treatment restored normal tissue histology, although proliferation of bile duct cells and dilatation of the portal vein were still observed (Fig. 8e). Quantitative assessment using Heijnenʼs score confirmed that the histopathology induced by AC (score of 126.4% vs. control) was markedly reduced by Cur. (by 34.01% compared to the AC group) and by N.Cur. (by 50.3% compared to the AC group) (Fig. 8f).

### Cur. and N.Cur. protected mouse liver from AC-induced liver fibrosis and glycogen depletion

**Figure 9:**
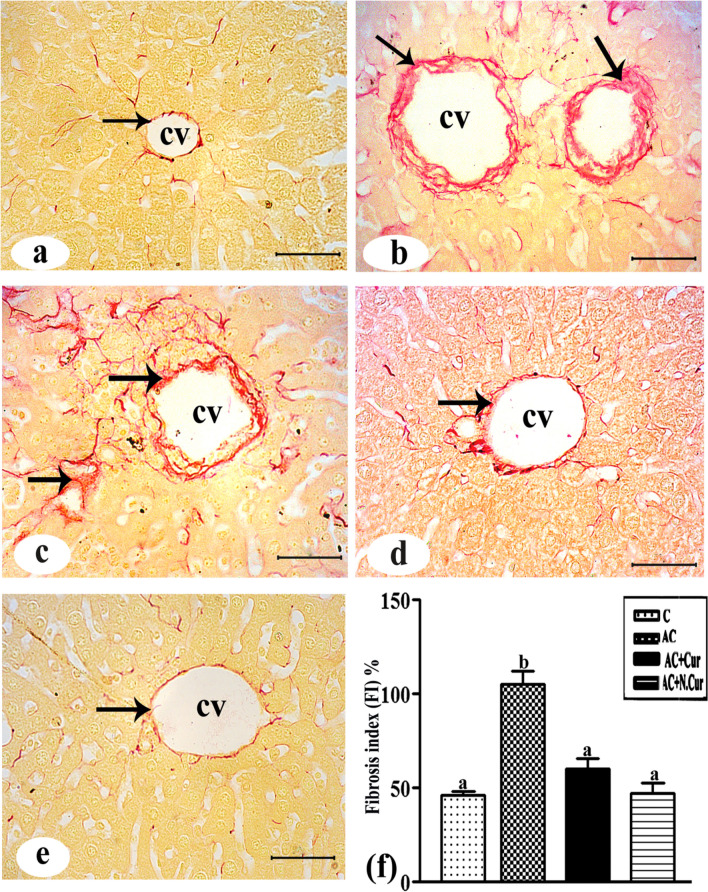
**(a**) High magnification image of liver tissue from a control male mouse showing few collagenous fibers around hepatocytes (arrow) or the central vein (cv). **(b and c)** Liver tissue from an AC group mouse showed large amounts of collagenous fibers (arrows) among hepatocytes and around the cv. **(d)** Liver tissues from AC + Cur and **(e)** AC + NCur group mice showing minimal amounts of collagenous fibers (arrow) around the cv (picrosirrus stain Bar = 50 μm). **(f)** Percentage liver fibrosis scores. Columns with different letters are significantly different (*p* < 0.001).

**Figure 10:**
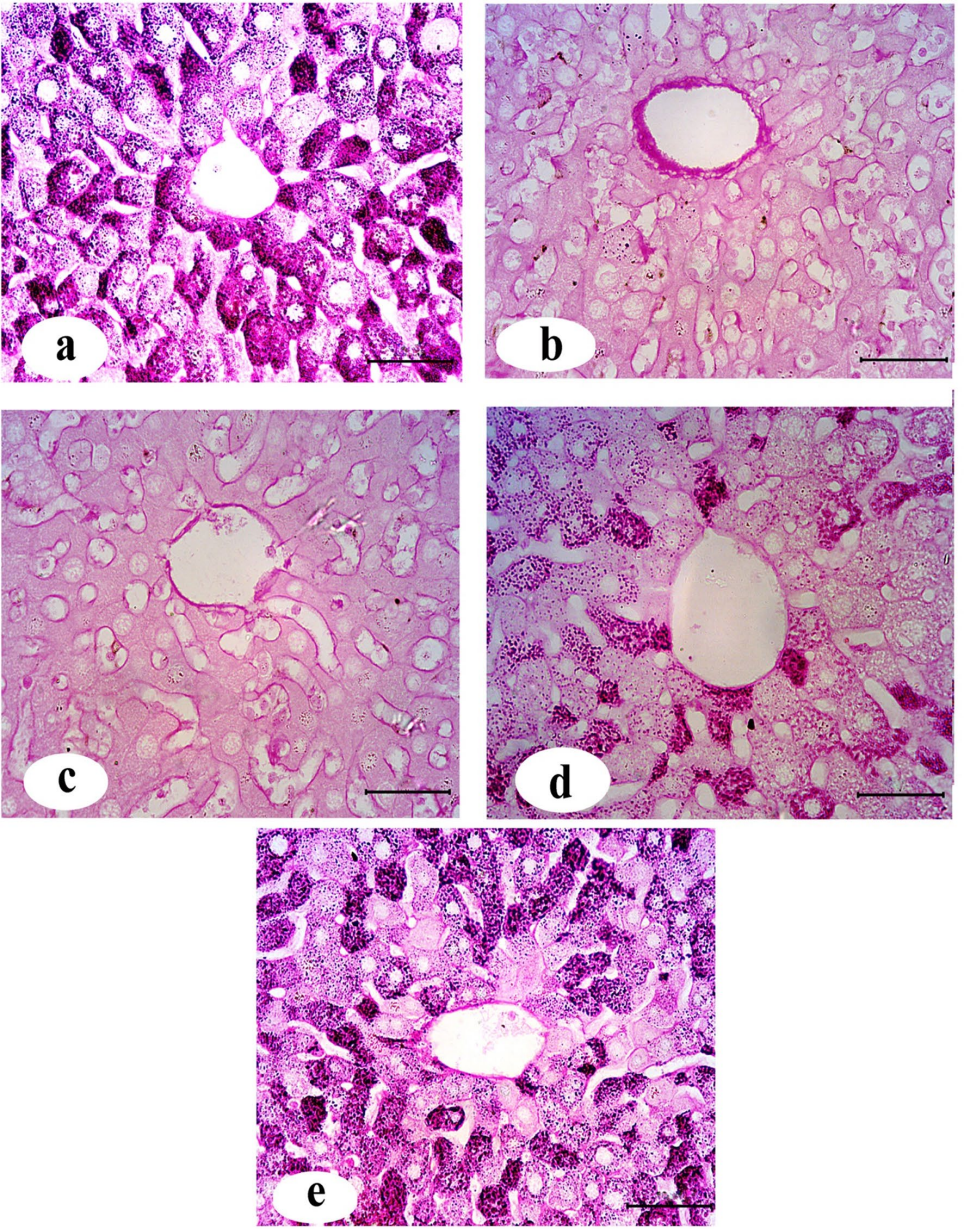
**(a**) High magnification image of liver from a control dense glycogen deposits. **(b and c)** AC group, showing marked glycogen depletion. **(d)** AC + Cur group, showing improved glycogen contents. **(e)** AC + NCur group, showing near-control levels of glycogen (PAS stain Bar = 50 µm).

Picrosirius red staining of liver sections from control mice revealed normal appeared collagen fibers (Fig. 9a), while AC treatment induced marked collagen fiber accumulation among hepatocytes and surrounding dilated and congested blood sinusoids (Fig. 9b). Cur. pretreatment reduced the number of collagen fibers surrounding hepatocytes, the central vein, and blood sinusoids (Fig. 9c) while N.Cur. appear to reduce collagen fiber density even further (Fig. 9d). Morphometric analysis of collagen deposition confirmed these qualitative observations, with AC increasing deposition by 126.9% compared to control mice (Fig. 9e) and both Cur. and N.Cur. reducing deposition by 42.9% and 55.0%, respectively, compared to the AC group. The rich distribution of red-stained glycogen particles in control liver (Fig. 10a) was markedly reduced in AC-treated liver (Fig. 10b), while both Cur. and N.Cur. treatment restored these glycogen stores (Fig. 10c and d).

## Discussion

In the current work, nanotechnology was used to break curcumin into nano-sized particles for increased bioavailability and enhanced membrane permeability. Acrylamide significantly increased the proliferation and viability of HepG2 and Huh-7 hepatic cancer cells while reducing apoptosis rate, while co-treatment with Cur. or N.Cur. increased apoptosis and mitigated the proliferative effects of AC. Further findings provided additional support for the antioxidant, immunomodulatory, and anti-inflammatory properties of nano-curcumin^33^.

There are many pathways involved in apoptosis induction, including the death receptor-dependent (extrinsic) and mitochondria-dependent (intrinsic) pathways^34^. While curcumin prevented AC-induced apoptosis, in accord with a previous study^35^, it had a little inhibitory effect on the proliferation of HepG2 cells, likely due to its poor water solubility^36^. In contrast, nano-curcumin exhibited a potent cytotoxic effect in both hepatic cancer cell lines tested and a greater antiproliferative effect than curcumin, likely due to enhanced cellular uptake as demonstrated by^37^. Curcumin has been shown to reduce ROS generation in various cell lines, which may account for reduced cell proliferation^38^.

Overexpression of CYP450, P53, and cleaved caspase-3 in response to AC may enhance tissue sensitivity to AC toxicity. Cur. significantly inhibited AC-induced CYP2E1 overexpression in the liver possibly via free-radical scavenging and antioxidant activities^4^. The p53 protein plays a central role in eliciting cellular responses to DNA damage, hypoxia, and aberrant proliferative signals, such as oncogene activation^39,40^, and nano-curcumin also significantly reduced the AC-induced increase in hepatic p53 expression, in agreement with previous reports^41^.

Acrylamide-induced toxicity is linked to oxidative stress, and long-term exposure to AC can induce mitochondrial dysfunction and apoptosis^42^. Activation of the mitochondria-mediated intrinsic apoptosis pathway was enhanced by nano-curcumin in HepG2 cancer cells in our finding may be the result of up regulation of pro-apoptotic Bax, down regulation of anti-apoptotic Bcl-2, and promotion of cytochrome c release from mitochondria^43^. Conversely, nano-curcumin significantly decreased cleaved caspase-3 in AC-treated liver, consistent with the observed antiapoptotic activity. Basniwal et al. investigated the effect of curcumin nanoparticles and their anticancer activities in cancer cell lines from the lungs (A549), liver (HepG2), and skin (A431). In aqueous circumstances, curcumin nanoparticles were found to have a far better effect on cancer cells than native curcumin^44^.

Sun et al. found that curcumin solid lipid nanoparticles (CUR-SLNs) exhibited increased cell uptake and growth inhibition in cancer cells, as well as better drug dispersibility and chemical stability^45^. In breast adenocarcinoma cells, CUR-SLNs were tested for antitumor efficacy (MDA-MB-231). In comparison to native curcumin, CUR-SLNs had higher solubility, biocompatibility, and toxicity. Furthermore, CUR-SLNs triggered cancer treatment by inducing considerably greater apoptosis in MDA-MB-231 cells^46^. Thus, nano-curcumin has dramatic reciprocal effects on cancerous and healthy hepatic cells, suggesting clinical promise as an anticancer agent without associated side effects due to non-target toxicity^43^.

Curcumin and nano-curcumin also reversed the AC-induced increase in COL1A1 mRNA expression. According to^47^, AC may induce hepatic gene expression abnormalities that result in the accumulation of collagen, a major pathological feature in a variety of liver disorders. A previous study has also found that curcumin and nan-ocurcumin can reduce collagen deposition^48^, suggesting therapeutic utility in fibrotic liver diseases.

Administration of AC also induced significant increases in plasma AST and ALT, liver-specific enzymes that are released from damaged hepatocytes and thus serve as biomarkers of hepatocellular injury. This finding may be explained by previous studies showing that AC increases hepatocyte membrane permeability and induces cellular transformation^41^, responses that may be attributed to the bipolar nature of AC (hydrophobic interactions and hydrogen bonds)^49^. The ability of curcumin and nanocurcumin to enhance endogenous antioxidant activity, resulting in reduced lipoperoxide formation^50^, may also contribute to the reductions in AST and ALT release.

Acrylamide administration caused degeneration, necrosis, hyperemia in interstitial vessels, cell vacuolation, nuclear pyknosis, and inflammatory cell infiltration in mouse liver. These hepatocyte vacuoles may protect hepatocytes^49^ by sequestering injurious substances and preventing them from interfering with biological activities. However, Mahmood et al. (2015) reported that AC can interfere with liver detoxification and excretion of toxic materials^51^.

The protective effects of curcumin against AC-induced damage have been attributed mainly to its antioxidant and radical scavenging properties^4^. However, curcumin may also have immunomodulatory activity, and acrylamide increased hepatic inflammation, in accord with^52^, who reported inflammation in multiple organs, as well as elevated neutrophil counts following AC treatment^25^ also reported that the bioavailability and controlled release of nano-curcumin could be responsible for increased cellular immune responses.

Many cells in the AC-treated liver demonstrated a weak PAS reaction indicative of reduced glycogen content, which suggests that AC induces glycogen breakdown into glucose^53^. Co-treatment with nano-curcumin completely restored glycogen content, suggesting that nano-curcumin could be a potential therapeutic agent for sustaining metabolic integrity under stress.

## Conclusion

**Figure 11:**
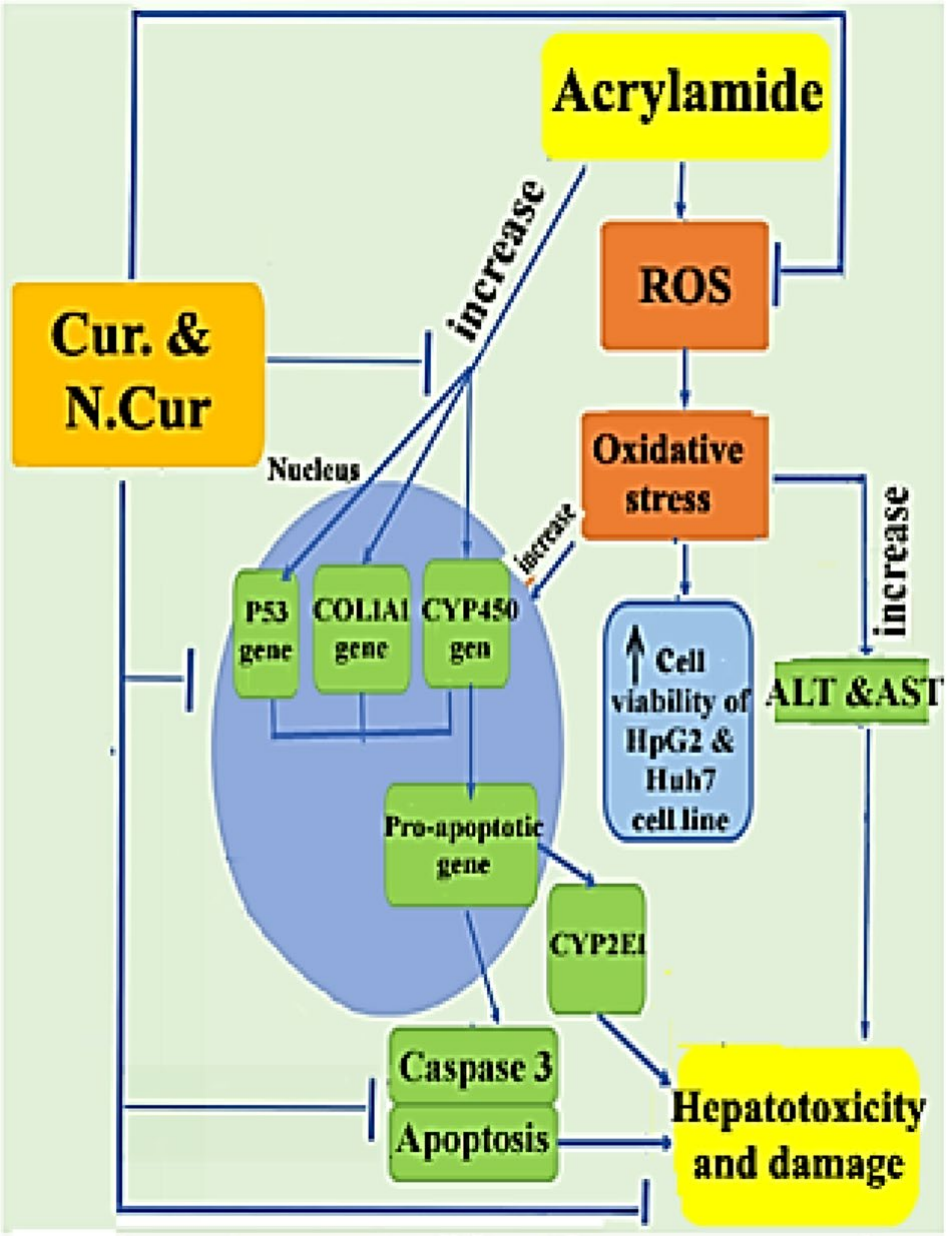
Graphical abstract shows the potential protection Cur. and N.Cur against acrylamide-induced toxicity, and oxidative stress via histopathological, biomarker, and molecular mechanism of some proteins in liver. Additionally demonstrates its cytotoxicity efficiency against HepG2 and Huh7 cell lines.

Collectively, these findings support the application of nano-curcumin for combating oxidative damage to hepatic cells induced by AC and down-regulating genes involved in fibrosis pathways, thereby preserving liver function and preventing fibrotic disorders. In addition, nano-curcumin may also be an effective therapeutic agent against hepatic cancer without the non-target toxicity of many other anticancer agents (Fig. 11).

## Supplementary Information

Supplementary Information.

## Data Availability

All data generated or analyzed during this study are included in this published article [and its supplementary information files].

## Abbreviations

AC: Acrylamide
Cur.: Curcumin
ROS: Reactive oxygen species
N.Cur: Nanocurcumin
EGTA: Ethylene glycol tetraacetic acid
TEM: Transmission electron microscopy
COL1A1: Collagen Type I Alpha 1 Chain
CYP2E1: Cytochrome P450 Family 2 Subfamily E Member 1
ALT: Alanine aminotransferase
AST: Aspartate aminotransferase
