# Supplementary figures and images for: Nanocurcumin and curcumin prevent N, N'-methylenebisacrylamide-induced liver damage and promotion of hepatic cancer cell growth
Source: Sci Rep. 2022 May 18;12:8319. doi: 10.1038/s41598-022-12406-y (PMC9117224; doi:10.1038/s41598-022-12406-y)

## The origin of western plot

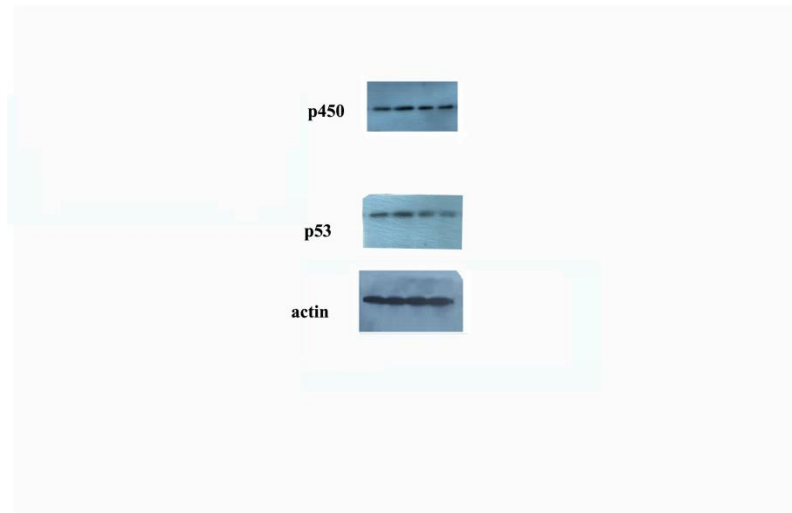

Supplement: Supplementary file 1 — Supplementary Information. [file 41598_2022_12406_MOESM1_ESM.pdf]
